# Evidencing the challenges of care delivery for people with intellectual disability and epilepsy in England by using the Step Together toolkit

**DOI:** 10.1192/bjo.2024.749

**Published:** 2024-10-28

**Authors:** Tom Shillito, Lance Watkins, Hafsha Ali, Georgia Page, Angie Pullen, Sarah Mitchell, Ashok Roy, Arjune Sen, Michael Kinney, Rhys Thomas, Phil Tittensor, Manny Bagary, Arun Subramanium, Bridie Kent, Rohit Shankar

**Affiliations:** Epilepsy Action, UK; University of South Wales, UK; Cornwall Intellectual Disability Equitable Research (CIDER), University of Plymouth Peninsula School of Medicine, UK; and Adult Learning Disability Epilepsy Service, Swansea Bay University Health Board, UK; NHS Midlands and Lancashire Commissioning Support Unit, Leyland, UK; School of Nursing and Midwifery (Faculty of Health), University of Plymouth, UK; Cornwall Intellectual Disability Equitable Research (CIDER), Cornwall Partnership NHS Foundation Trust, UK; Adult Learning Disability Service, Coventry and Warwickshire Partnership NHS Trust, UK; Oxford Epilepsy Research Group, Nuffield Department of Clinical Neurosciences, John Radcliffe Hospital/University of Oxford, UK; Neurology Services, Belfast Health and Social Care Trust, UK; Translational & Clinical Research Institute, Newcastle University, UK; Neurology Services, The Royal Wolverhampton NHS Trust, UK; Neuropsychiatry Services, Birmingham & Solihull Mental Health Trust, UK; Adult Learning Disability Services, The Southern Health and Social Care Trust, UK; Cornwall Intellectual Disability Equitable Research (CIDER), University of Plymouth Peninsula School of Medicine, UK; School of Nursing and Midwifery (Faculty of Health), University of Plymouth, UK; and Cornwall Intellectual Disability Equitable Research (CIDER), Cornwall Partnership NHS Foundation Trust, UK

**Keywords:** Seizures, systems, care planning, quality improvement, developmental disorders and neurodevelopment.

## Abstract

**Background:**

People with intellectual disability (PwID) and epilepsy have increased premature and potentially preventable mortality. This is related to a lack of equitable access to appropriate care. The Step Together guidance and toolkit, developed with patient, clinical, charity and commissioning stakeholders, allows evaluation and benchmarking of essential epilepsy service provision for PwID in eight key domains, at a care system level.

**Aims:**

To evaluate care provisions for adult PwID and epilepsy at a system level in the 11 integrated care systems (ICSs) of the Midlands, the largest NHS England region (population: approximately 11 million), using the Step Together toolkit

**Method:**

Post training, each ICS undertook its benchmarking with the toolkit and submitted their scores to Epilepsy Action, a national UK epilepsy charity, who oversaw the process. The outcomes were analysed descriptively to provide results, individual and cumulative, at care domain and system levels.

**Results:**

The toolkit was completed fully by nine of the 11 ICSs. Across all eight domains, overall score was 44.2% (mean 44.2%, median 43.3%, range 52.4%, interquartile range 23.8–76.2%). The domains of local planning (mean 31.1%, median 27.5%) and care planning (mean 31.4%, median 35.4%) scored the lowest, and sharing information scored the highest (mean 55.2%, median 62.5%). There was significant variability across each domain between the nine ICS. The user/carer participation domain had the widest variation across ICSs (0–100%).

**Conclusions:**

The results demonstrate a significant variance in service provision for PwID and epilepsy across the nine ICSs. The toolkit identifies specific areas for improvement within each ICS and region.

## Intellectual disability

Intellectual disability (also known as learning disability in UK health services) is a neurodevelopmental disorder characterised by deficits in both cognitive and adaptive function across practical, social and conceptual domains.^1^ People with intellectual disability (PwID) are not a homogenous group; the more severe the impact on cognitive and functional domains, the higher the burden of comorbid medical conditions, including epilepsy. Approximately 2% of the UK population meet the diagnostic criteria for intellectual disability.^2^ The multimorbidity rates for PwID are very high (98.7%).^3,4^

## Epilepsy

Epilepsy is a neurological condition broadly characterised as an enduring predisposition to generate seizures.^5^ It affects around 1% of the UK population, and prevalence and incidence rates are highest in areas of deprivation.^6^ Life expectancy for people with epilepsy is at least 10 years lower than the general population.^7,8^ There is evidence that epilepsy mortality rates are not improving over time compared with other chronic health conditions.^9^

## Epilepsy and intellectual disability

Almost a quarter of PwID have epilepsy (22%),^10^ and around two-thirds will have treatment-resistant seizures.^11^ Epilepsy is also one of the most common reasons for avoidable hospital admissions in this group, with 40% of all emergency admissions for PwID attributable to seizures.^12^ The risk of death for PwID and epilepsy is up to ten times higher than for those with intellectual disability who do not have epilepsy.^10^ Epilepsy is a common comorbidity for PwID, and it significantly increases their risk of mortality.^13–15^ Table 1 gives more information on the impact epilepsy and intellectual disability can have on this population.

**Table 1:** The synchronous relationship between epilepsy and intellectual disabilities

Intellectual disabilities
Intellectual disability (or learning disability) is a neurodevelopmental disorder characterised by deficits in both cognitive and adaptive function across practical, social and conceptual domains.^1^ 2% of the UK population meet the diagnostic criteria for intellectual disability.^2^ The multimorbidity rates for PwID are very high (98.7%) ^3,4^
Epilepsy and intellectual disabilities
Epilepsy is a neurological condition broadly characterised as an enduring predisposition to epileptic seizures.^5^ Affects 1% of the UK population, and prevalence and incidence rates are highest in areas of deprivation.^6^ Almost a quarter of PwID have epilepsy (22%),^10^ and around two-thirds will have treatment-resistant seizures.^11^ Epilepsy is also one of the most common reasons for avoidable hospital admissions, with 40% of all emergency admissions for PwID attributable to seizures.^12^
Mortality
Epilepsy and intellectual disability both affect mortality, and when they co-occur, this effect is significantly increased.^13^ Life expectancy for people with epilepsy is at least 10 years lower than the general population.^7,8^ Epilepsy mortality rates are not improving over time, unlike other chronic health conditions.^9^ In a 2020–2021 audit of UK deaths of PwID, epilepsy was found to be associated with a third of the deaths reviewed; 49% of deaths in this group were avoidable, compared with 22% of the general population.^13^ The risk of death has been found to be up to ten times higher for this group compared with PwID who do not have epilepsy.^10^ Sudden unexpected death in epilepsy, one of the most common causes of death for people with epilepsy, is up to nine times higher for PwID than those without.^14^ Mortality rates are 13 times higher for individuals with more severe intellectual disability.^15–17^

PwID, people with intellectual disability.

## Step Together

A 2018 report by the British Branch of the International League Against Epilepsy (ILAE) Working Group on services for adults with epilepsy and intellectual disability, highlighted the problems with fragmented care and poor risk management for this group within the National Health Service (NHS).^16^ This report also raised issues with inequitable access to care for this group, and a need for support and guidance for healthcare professionals who support this group, but do not work within intellectual disability.

In the absence of nationally agreed guidance or structures for epilepsy reviews for PwID, vital aspects of care can slip through the gaps in service and communication.^11,17–19^ This may be contributing to the significantly higher mortality seen in this group.^13^ PwID with epilepsy have a high prevalence of multimorbidity, and will have clinical contact with a range of professionals.^3,4^

There is a need for multi-speciality collaborative working to ensure high-quality care.^20^ An integrated service model offers an opportunity to provide holistic care.^20–22^ This led to the Step Together project, which proposed an evidence-based and validated model to examine and provide best practice for managing epilepsy in PwID, and help create capable communities.^23,24^ The model was co-created by a wide range of health and social care professionals and PwID and epilepsy. Full details of the creation and validation of the model can be found in Appendix 2. This model has been identified as best practice to support Learning Objective 6.1 in the ILAE Curriculum.^20^ Further details of Step Together and capable communities proposed is provided in Appendices 1 and 2. The Step Together toolkit is focused on care for adults in English healthcare systems, and can be completed at both service and system levels. It benchmarks the service and system, and allows easy comparison between services and systems and across time.

## Integrated care systems

To reduce regional inequalities in England, in July 2022, the NHS and its collaborators (social care, voluntary sector, etc.) moved to a new systems model called integrated care systems (ICSs).^25^ There are 42 ICSs in all of England organised into six regions, which are local partnerships that bring health and care organisations together to develop shared plans and joined-up services.^25^

## Aim

The primary objective was to examine the care provision for PwID and epilepsy across different ICSs, using the Step Together toolkit (Supplementary File 1 available at https://doi.org/10.1192/bjo.2024.749). The secondary objective to gain system-level feedback of the utility of the Step Together toolkit by the participating ICSs.

## Method

### Eligible population

The UK Midlands region is home to about a fifth of the population of England (approximately 11 million).^26^ The number of people living with epilepsy in the Midlands is estimated to be 95 000,^6^ and the number of people with an intellectual disability in the Midlands is estimated to be 230 000 (calculated from 2.17% of the UK population having intellectual disability).^27^ The estimated number of people with both epilepsy and intellectual disability in the Midlands is 20 900 (calculated from 22% of people with epilepsy having intellectual disability^10^).

### The Step Together toolkit

The Step Together toolkit (Supplementary File 1), based on the Step Together guidance,^23^ is a self-assessment tool that can be used by services, NHS Trusts, English healthcare systems and regions across England, to understand and evaluate the care they provide to adults with both epilepsy and an intellectual disability. It is hosted by the national UK charity Epilepsy Action (https://www.epilepsy.org.uk/professional/step-together#row-fc-4).Details of the development and validation of the toolkit can be found in Appendix 2.

It is a quantitative tool made up of 56 questions, covering eight domains:

- Workforce: covering access to healthcare professionals, ease of recruitment and retention, presence of named leads for quality improvement, and workforce strategies.
- Local planning: covering commissioning of services, provision agreements, joint meetings between services, and clinical pathways.
- Key service provision: covering primary care and social care involvement, agreements between services regarding shared care and care provision, joint care planning and case-loads.
- Diversity of provision: covering voluntary sector involvement and representation.
- Care planning: covering care plans and hospital passports, sharing of care plans between services, access to care plans and patient understanding of care plans.
- Transition: covering transition from paediatric to adult care and reasonable adjustments.
- Sharing information: covering access to local services, sharing of patient records between services, and support services.
- Patient and carer participation: covering patient and carer/family involvement in decision making at a service level, ability to provide feedback and receive a response, and carers’ needs assessments.

The majority of questions are ‘yes/no’ or Likert scale questions. Each question response is assigned a score. These scores are used to calculate a percentage score overall and individual percentage scores for each of the eight constituent domains. Overall and domain-specific scores are RAG rated, with results below 30% rated red, between 30 and 70% rated amber and over 70% rated green. Red indicates that there is a need for significant improvement across many areas (either within the individual domain being scored, or across many domains). Green indicates that most minimum requirements are being exceeded and services are generally performing well. Amber indicates a need for improvement in some areas, where either some minimum requirements are not being met, or many minimum requirements are being met but very few are being exceeded. A perfect score of 100% would show that all areas of care for PwID and epilepsy are being provided to the highest quality possible.

### Study design

With the support of NHS England (Midlands), the 11 ICSs in the Midlands were approached and asked to complete the Step Together toolkit at a system level.

Initial conversations were held with senior members of each ICS, and online webinars were set up to provide the Step Together background, relevance and how to use the toolkit to generate interest and buy-in. The webinars were attended by representatives of all levels of the ICS and services. Additional online training webinars were held to explain the toolkit completion process to those involved. Each ICS was also asked to hold their own internal events to inform relevant stakeholders about the toolkit completion process.

Each ICS was asked to nominate a Step Together champion, whose role was to support the completion of the benchmarking, be responsible for collating the data of their system and assemble the final response.

This champion distributed the relevant questions to each team/individual, arranged feedback sessions and collated the final toolkit response. It was recommended that the champion spoke to relevant staff within neurology, paediatrics, intellectual disability services, social care, voluntary sector partners, commissioners and, where possible, patients and their families and carers. Drop-in clinics were made available for champions to consult the study team on any aspect of information gathering on the tool.

Each toolkit response was then analysed to give a set of results for the individual ICS. The results were compared between ICSs to give a region-wide perspective of care for people with epilepsy and intellectual disability in the Midlands. Areas of good practice and areas for improvement were identified based on the toolkit responses, both for individual ICSs and the region. Scores were calculated for the overall Midlands region by taking the mean responses of each individual ICS result. An overall score and individual scores across the eight constituent domains were calculated.

### Ethics and governance

This study does not report on any human participants, but on the systems their data is collected in. In addition, each ICS Step Together champion registered the data collection tool as an audit/service evaluation in their region and undertook Data Protection Impact Assessments in line with local policies. Approval was gained by each ICS site from local information governance teams. Only de-identified data was submitted to the central data-set held by Epilepsy Action. The transfer was in compliance with the General Data Protection Regulation. This study did not require formal ethical approval as it did not directly deal with patients. Epilepsy Action UK and NHS England Midlands oversaw the project governance (Supplementary File 2). Additional confirmation that no NHS ethics is required was obtained (Supplementary File 3).

## Results

The toolkit was completed by ten of the 11 Midlands ICSs between November 2022 and February 2023 (Table 2). The population covered by each ICS varied from 806 534 to 1 577 949 people. Those ICSs with less than a third of the population in the lowest two deprivation index deciles were associated with higher number of general practitioners (GPs) per 100 000 people and lower number of LeDeR reported annual deaths associated with epilepsy for 2022–2023. The full details of each ICS population, deprivation indices, GPs per 100 000 people and LeDeR deaths in epilepsy reported is provided in Table 2.

**Table 2:** Results: demographics for each participating integrated care system in the Midlands region

Integrated care system	Population^28^	Estimated number of people with epilepsy and an intellectual disability^a^	% of population in lowest two IMD quintiles^b^,^29^	General practitioners per 100 000 people^30^	Number of LeDeR deaths in 2021–2022^c^	Case-load of people with epilepsy and an intellectual disability given in toolkit response
1	818 249	1596	27.1	68.95	18	100
2	1 240 698	2419	46.2	62.96	81	550
3	806 534	1573	38.6	51.45	86	1307
4	1 111 009	2166	36.4	63.29	7	17
5	1 052 979	2053	32.2	64.75	69	Not provided
6	1 277 444	2491	65.7	58.31	81	258
7	1 172 053	2286	37.4	57.32	70	1190
8	1 577 949	3077	69.2	65.22	81	Not provided
9	1 185 265	2311	30.5	62	73	65
10	814 554	1588	33.2	61.64	26	500 (paediatrics only)

a. Estimated using the population size, epilepsy prevalence of 0.09^6^ and prevalence of intellectual disability in the epilepsy population of 0.2.^10^

b. IMD is Index of Multiple Deprivation (IMD), which is a measurement of deprivation based on multiple measures recorded by local government. These include employment, healthcare, income and housing. The lowest quintile has the highest level of deprivation.

c. LeDeR death data for each region were collected from the local LeDeR reports:

Nottingham & Nottinghamshire CCG, LeDeR Programme 2021–2022.^31^

Lincolnshire Integrated Care Board, Annual Report for the LeDeR Programme in Lincolnshire 2021–2022.^32^

LeDeR Annual Report for Derby and Derbyshire 2021–22.^33^

Coventry and Warwickshire Integrated Care Board, LeDeR Programme Annual Report 2021–2022.^34^

Black Country and West Birmingham Clinical Commissioning Group, LeDeR Annual Report 2021–2022.^35^

Staffordshire and Stoke-On-Trent Integrated Care System, LeDeR Programme Annual Report 2021–2022.^36^

Birmingham and Solihull Annual LeDeR Report 2021–22.^37^

Leicester, Leicestershire and Rutland Clinical Commissioning Groups, LeDeR Annual Report June 2022.^38^

Northamptonshire LeDeR Annual Report 2022/2023.^39^

The scores for the Midlands region were based on the responses given by adult services from nine of the 11 ICSs in the region. One ICS in the Midlands did not submit a toolkit response. One ICS submitted a toolkit response only for their paediatric services. The toolkit was designed primarily for use by adult services (other than the section on transition), so the paediatric results have not been included in the overall score.

**Fig. 1:**
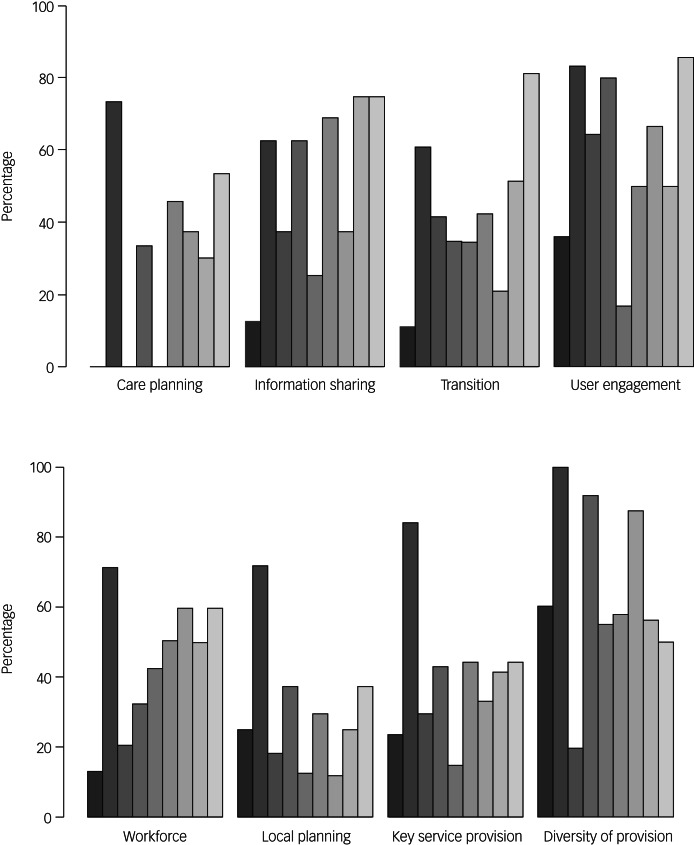
Step Together toolkit scores for each integrated care system in the Midlands region, across the eight domains assessed (*n* = 9).

The Midlands region had an overall score of 44.2% (mean 44.2%, median 43.3%, range 52.4% (minimum 23.8%, maximum 76.2%). In the RAG rating used within the toolkit, this puts the Midlands region in the amber category, where many services are adequate but there is scope for improvement in many areas. Table 3 and Fig. 1 show the scores for each of the nine individual ICSs across all eight domains. They are summarised below. They key outcomes from the ICSs are summarised in Table 4. A summary of the recommendations made to the region based on their results can be seen in Table 5.

**Table 3:** Mean, median and range of responses from all systems across each of the domains

Domain	Mean, %	Median, %	Range, % (minimum %, maximum %)
Workforce	38.2	46.3	60.7 (13.3, 74)
Local planning	31.1	27.5	71.9 (0, 71.9)
Key service provision	38.1	38.8	69.3 (14.8, 84.2)
Diversity of provision	45.8	57.1	80.0 (20, 100)
Care planning	31.4	35.4	73.3 (0, 73.3)
Information sharing	55.2	62.5	75.5 (12.5, 88.0)
Transition	40.0	38.8	69.8 (11.1, 81.0)
User engagement	42.7	57.1	100 (0, 100)
Total	44.2	43.3	52.4 (23.8, 76.2)

Some small discrepancies may result from rounding totals.

**Table 4:** Impact: key outcomes identified by the systems following toolkit completion

Increased focus on improving epilepsy care for people with an intellectual disability and/or autism embedded in system-wide improvement to tackle health inequalities. Bringing together of system partners across primary, secondary and social care, and voluntary sector to drive improvement. Shared understanding of the extent to which services are working well together to deliver good care. Shared understanding of where gaps in provision or quality exist, and springboard for new or enhanced services identified. Shared understanding of workforce capacity and capability, and where further investment is needed. Better use of system-wide resource to improve outcomes. Foundations in place to support the development of integrated epilepsy care. Improved integrated strategic commissioning of epilepsy services and support.

**Table 5:** Recommendations for the Midlands regions based on their Step Together toolkit results

Systems should work together to address challenges in recruiting and retaining staff. This could include joint training programmes to upskill staff currently in the region, region-wide recruitment schemes and incentives for staff to remain in the region when changing roles The number of people with intellectual disability and epilepsy in their catchment area should be known by every integrated care system (ICS). Measures should be taken to ascertain this data, and processes put into place to update it regularly. This data should be used to inform commissioning of services for this group Mechanisms to allow greater participation of patients with intellectual disability and epilepsy in decision-making at a service and ICS level should be introduced and promoted. Patients should be contributing to the design and delivery of the services they use. Regional patient forums should be used to monitor the consistency of care between services and ICSs. Single joint care plans covering both epilepsy and intellectual disability should be introduced. Shared care agreements and protocols should be introduced in each ICS, to ensure all aspects of care are covered between the intellectual disability and epilepsy services. Every ICS should have a named commissioner responsible for care for people with intellectual disability and epilepsy. This could be combined with the role of the person responsible for LeDeR oversight (monitoring deaths of people with intellectual disability). Having an individual in each ICS with a similar role would allow for ICSs to share concerns and problem solve on a regional level, and allow for sharing of best practice between ICSs. Links between health and social care services, local councils and voluntary sector services should be strengthened to encourage holistic care across the whole spectrum of support available. ICSs should complete the Step Together toolkit or a similar service evaluation regularly (every 3–5 years) to monitor changes over time.

### Results by domain

#### Workforce

Systems in the Midlands generally found it difficult to hire and retain suitable staff to support people with epilepsy and an intellectual disability.

All nine ICSs were providing access to all or most (at least 66%) of the recommended healthcare providers (see toolkit in Supplementary File 1 for list) (mean 80%, minimum 66%, maximum 100%). Six of the nine ICSs (67%) did not feel that the severity of intellectual disability had a substantial impact on a patient's ability to access services.

However, eight of the nine ICSs (89%) felt that it was very difficult to recruit and retain staff with the skills and knowledge required to support people with an intellectual disability and epilepsy. The one remaining ICS (11%) felt it was moderately difficult. Few ICSs had agreed cross-agency workforce strategies and action plans. Seven ICSs (78%) either disagreed or strongly disagreed that one was in place, and the remaining two ICSs agreed, but did not strongly agree. Six ICSs (67%) did not have a named lead in both epilepsy and intellectual disability services responsible for ensuring regular multidisciplinary care of patients. Two ICSs (22%) did have named leads, and one ICS (11%) had named leads in half of the Trusts within it.

#### Local planning

Local planning is a significant issue for most ICSs in the Midlands. This domain received the lowest mean (31.1%) and median (27.5%) scores.

Five ICSs (56%) commissioned services for PwID with epilepsy separately from general epilepsy commissioning. Four ICSs (45%) felt the service provided for PwID and epilepsy had clear specific clinical pathways, and one ICS (11%) felt this was true for half of their Trusts.

However, seven ICSs (78%) did not have a named commissioner responsible for intellectual disability and epilepsy. One ICS (11%) had an agreement between intellectual disability and epilepsy services relating to commissioning roles, and no ICSs had an agreement relating to role provision. There was zero assurance of the capability and competency of residential care providers and hospitals in delivering safe in-patient epilepsy care in any of the ICSs (two ICSs neither agreed nor disagreed that this had been received, all other ICSs disagreed or strongly disagreed). Only three ICSs (33%) had identified the number of PwID with epilepsy and identified their needs to inform service plans.

#### Key service provision

Ethnicity was routinely recorded in two-thirds of the ICSs (67%). Most ICSs felt there was sufficient training for staff to understand behavioural and cultural issues (five agreed, two neither agreed nor disagreed). Primary care registers for PwID existed in six ICSs (66%), and annual health checks were performed in all ICSs. Most ICSs always or sometimes had joint care planning at an individual level for patients accessing both epilepsy and intellectual disability services (one always had it (11%), six often had it (67%), two never had it (22%)).

However, no ICSs had agreed measures and monitoring of different pathways/services in epilepsy and intellectual disability services to ensure consistency of care. Only one ICS (11%) had protocols for the transfer of shared care between epilepsy and intellectual disability services. Only one ICS used joint care plan documents that specified what would be provided by epilepsy and intellectual disability services. Seven ICSs (78%) did not have formal agreements between intellectual disability, epilepsy and primary care services about the responsibilities of primary care staff and referral routes for specialist support or discharge back from specialist care.

A consistent finding was the poor identification systems to capture the number of PwID and epilepsy in each system. Only two ICSs came close to the estimated prevalence of PwID and epilepsy for their region (Table 2).

#### Diversity of provision

Six ICSs (67%) had a health and well-being promotion strategy that included and adequately addressed the requirements of PwID, and one ICS (11%) without a strategy had work underway to integrate promotion into practice. However, most ICSs (six, 66%) felt there were not sufficient voluntary sector services that supported people with epilepsy and intellectual disability. Some ICSs also felt the voluntary sector services that were present struggled to support PwID (four agreed or strongly agreed (44%), two disagreed or strongly disagreed (22%), three neither agreed not disagreed (33%)).

#### Care planning

This was another domain that received very low scores (mean 31.4%, median 35.4%). There was significant variation across the ICSs (range: 73.3%).

Four ICSs (44%) agreed that every patient with epilepsy and intellectual disability, and their carers, have a clear understanding of their care plan and the services available for routine and emergency care. However, only three ICSs (33%) had single care plans shared between epilepsy and intellectual disability services. Six ICSs (66%) did not have care plans that could be accessed 24 h a day by staff providing direct care across different services.

#### Transition

There was significant variation between ICSs’ self-assessment of transition from child to adult services. Six ICSs (66%) agreed or strongly agreed that reasonable adjustments were made to meet the needs of PwID when transitioning to adult services. However, four ICSs (44%) disagreed or strongly disagreed, and three (33%) neither agreed nor disagreed that their transition arrangements were good, with clear information and transparent policies, provided well in advance of the transition.

#### Sharing information

This domain received the highest mean (55.2%) and median (62.5%) scores, but there was significant variation in scores from each ICS (range 75.5%)

Five ICSs (56%) confirmed that patients and their families had access to clear guidelines on the management of emergencies that are reviewed as necessary. Epilepsy and intellectual disability records were shared well between primary, secondary (neurology) and intellectual disability care services in seven ICSs (78%). However, eight ICSs (89%) did not feel there was adequate support to meet patients’ needs around housing, welfare and benefits, meaningful activities and crisis plans, all of which could have an impact on their seizure management. Electronic health records were joined across specialist acute, primary care, intellectual disability and social care services in one ICS (11%), and partially joined in two other ICSs (22%). They were not joined at all in six ICSs (66%). Two ICSs (22%) had a comprehensive care directory of relevant local services that specifically work with people who have epilepsy and intellectual disability, which was available both to the public and to relevant healthcare professionals. Five ICSs (56%) did not have a directory, and two ICSs (22%) had a directory that was only available to certain healthcare teams.

#### Patient and carer participation

This domain had the widest variation across ICSs, with some ICSs scoring 0% and others 100%.

Seven ICSs (78%) had a mechanism for PwID and their carers to provide feedback and receive a response. However, seven ICSs (78%) did not have services where specifically PwID and epilepsy were making a significant contribution. One ICS (11%) did, and one ICS (11%) had work underway to establish this.

## Discussion

The NHS Constitution promises the same level of comprehensive care to all citizens across England.^40^ This should mean that a person with intellectual disability and epilepsy is provided the same services of similar standards across England. The results from the England Midlands region demonstrate significant variance in service provision between the ICSs included, indicated by the large range in results across all of the eight domains. Based on the findings, it can be asserted that there is a major lack of consistency in the service provision for people with epilepsy and intellectual disability. Furthermore, the lack of being able to identify the numbers in the ‘population at risk group’ by seven out of nine ICSs is concerning. NHS digital healthcare records should have a ‘reasonable adjustment flag’ to identify those with intellectual disability.^41^ There should be mechanisms to identify how many of those who have intellectual disability also have an epilepsy diagnosis. The lack of such basic data suggests it would be difficult even to comprehend the challenges and thus resource the concerns.

These findings are expected and consistent with previous reports outlining the concerns of fragmented care provision and lack of access to equitable care for this vulnerable group.^16^ However, such reports were developed largely on clinical and patient perceptions through cross-sectional surveys, and could be suspected to be having a respondent bias. However, the Step Together guidance and toolkit provides, for the first time, hard evidence of this fragmentation by benchmarking services. As has been mentioned holistic support is imperative to save lives in people with epilepsy and intellectual disability.^17^

The variation between systems is most likely a result of the significant variation in care seen in different ICSs. Service set-up, patient pathways and management structures are very variable across the UK.^16^ This was also seen in the workshops used to create the toolkit (Appendix 2). The toolkit was created to be adaptable to these variations.

### Utility of the toolkit

Implementing the toolkit with an ICS approach is complex, involving many stakeholders. The feedback received from the ICSs was positive. Specific feedback from four of the ICSs can be seen in Table 7. The coordinated feedback response of each ICS Step Together champions found the Step Together toolkit gave a useful overview of the strengths and weaknesses present across the ICS, as well as allowing for specific recommendations for improvement. The process of completing the toolkit brought together a wide range of partners who would not ordinarily communicate, and those conversations themselves highlighted gaps and missed opportunities to support this group. The regional report highlighted the strengths of neighbouring regions and prompted cross-ICS conversations to share learning and best practice. The aim of the toolkit in identifying areas of system strength to reinforce or protect them, and develop areas that are not as well established, was delivered.

The implementation of the toolkit has highlighted areas of best practice and areas for improvement within each individual ICS and the region (Tables 4 and 5). Specific examples include targeted investment in epilepsy specialist nurses, investment in other dedicated resources for the intellectual disability and epilepsy population, and focused collaboration between epilepsy specialist nurses and other professionals to improve integrated working and capability.

The use of the toolkit in the Midlands region has led to a number of improvements over a short period of time. This has shown the benefit of the toolkit and of benchmarking in this way (Tables 4, 6 and 7).

**Table 6:** Overall take home messages from Step Together toolkit implementation in the Midlands region

Key findings:
The Step Together toolkit was easy to use The toolkit gave feedback that was relevant and implementable The systems have reported ongoing improvements in care for people with epilepsy and intellectual disability following toolkit completion

**Table 7:** Feedback from four of the Step Together champions in the Midlands region on the use of the Step Together toolkit

‘To continue to improve capability and capacity for commissioners, epilepsy is now a distinct workstream in our LD/A planning. [Our] LeDeR Governance Panel approved ‘Epilepsy’ as a local variation for focussed LeDeR Reviews. The Task and finish Group [established to complete the toolkit] will become a forum with strategic oversight of pathway scoping and commissioning intentions for our citizens with intellectual disability and epilepsy. The initial webinar provided a platform to build on for the future, with [our] ICB now hosting quarterly webinars, with a shared focus on ‘hot topics’ from our LeDeR programme, commencing with SUDEP in Q1 2023/2024. These system commitments will help reduce the risk of health inequalities and premature mortality for our people with intellectual disability and epilepsy.’ ‘The toolkit cannot be completed without input from a very wide range of stakeholders, and even before the toolkits had been returned we could see the benefits those conversations were having. This approach required providers to be aware of and evaluate against the patient journey, the range of services this population need to support them from both a health and social care perspective, the number of people involved in their care, and the conflicting needs of each service. It also highlighted the needs this population require outside of each individual professional's speciality.’ ‘The process to undertake this self-assessment has created not only ownership of this issue but helped to create advocates for change. The product of all stakeholders being systematically involved in this work has enabled systems to collectively review and benchmark how all services can best meet the needs of this population; to identify gaps, variation, challenges and to focus their service improvement plans on agreed areas of impact for service users. ‘Client and stakeholder expectations have been exceeded. Recommendations to partners were specific and gave a clear steer where differences could be made for patients’ safety in a neutral non-judgemental fashion, providing a strong platform on which to go forward with a ‘can do’ purpose. This work has left us not only with quantifiable actions, made populations visible and given system ownership of the service issues faced in this critical area, but has created an advocacy for change and a social movement to improve the lives for whom we absolutely need to, and can, make a difference.’

LD/A, learning disability/autism; ICB, Integrated Care Boards; SUDEP, sudden unexpected death in epilepsy; Q1, Quarter 1 (i.e. January 1st to March 31st).

The Step Together toolkit was designed for use within adult services in the NHS. Although this paper has used it at an ICS level, it can also be used on a more granular scale (Appendix 2). There is also scope for the tool to be adapted for use in other healthcare systems. We recommend a similar process as that outlined in Appendix 2 is used to adapt the questions to reflect the realities of a different system.

### Limitations

The ICSs gave feedback on their experiences using the toolkit during and after the process. The most significant challenge to implementation of the toolkit within the NHS is the availability of staff with the relevant knowledge to complete it. It requires cooperation between many different teams and departments, including social care and, where possible, patient engagement. This can be very challenging in a healthcare system that is already overstretched. The impact of this was mitigated through the use of a coordinator for each system, who would organise distribution of relevant questions, arrange feedback sessions and assemble the final toolkit response. This allowed the toolkit to be completed in a timely manner across multiple departments. It also reduced the staff cost related to completion, as staff time was used efficiently by only sharing questions relevant to each individual/team, and with the coordinator tasked with resolving discrepancies between answers.

The toolkit is only as accurate as the responses entered. The ICSs were encouraged to take ownership of the toolkit completion process. This allowed them to see the toolkit as a tool for growth and change that would benefit the ICS. There is a risk that inaccurate answers could be input to make services appear better or worse than they are. It is thus strongly recommended that the toolkit is not used in a punitive manner, and that it is viewed as a quality improvement tool.

In conclusion, people with epilepsy and intellectual disability are at a significantly higher risk of mortality and reduced quality of life. They require ongoing personalised, holistic care. Improvements in care are needed to reduce the high numbers of avoidable deaths and hospital admissions in this group.

### Implications for clinical practice

The toolkit provided an evidence-based overview of where there is potential for fragility in service provision. It allows for services to be assessed in an easy way, that quickly highlights potential improvement options (Table 7). It can improve connectivity between teams/services, focus improvement plans on areas of need and improve ownership of improvement processes (Table 7). As done in the Midlands, it can be successfully and rapidly used to benchmark services within and across ICSs, and generate quality outcomes that can lead to improvement in care provision.

### Implications for policy

The toolkit can be used to compare regions and also revisit the same region at a different point of time. It provides a consistent approach to this. Further, it can be used to highlight key gaps in the ICS that could otherwise leave a vulnerable population at risk (Table 7).

### Implications for research

The toolkit collects a significant amount of data across systems and across time. This data could be analysed to understand patterns of care and potential associations with a host of clinical and social outcomes, including premature mortality. Further research to explore similar benchmarking toolkits for other conditions comorbid with intellectual disability should be explored. The utility of the toolkit needs could be further examined by comparing the results from the toolkit are related to any important outcomes for the ICS (e.g. deaths).

The toolkit could also be adapted for use in other countries’ healthcare systems, to allow for learning across borders. Together, there is an opportunity to build proactive big-data systems, which would lend itself to predictive machine learning approaches.

## Supporting information

Shillito et al. supplementary material 1 Shillito et al. supplementary material

Shillito et al. supplementary material 2 Shillito et al. supplementary material

Shillito et al. supplementary material 3 Shillito et al. supplementary material

Shillito et al. supplementary material 4 Shillito et al. supplementary material

## Figures and Tables

**Fig. 2:**
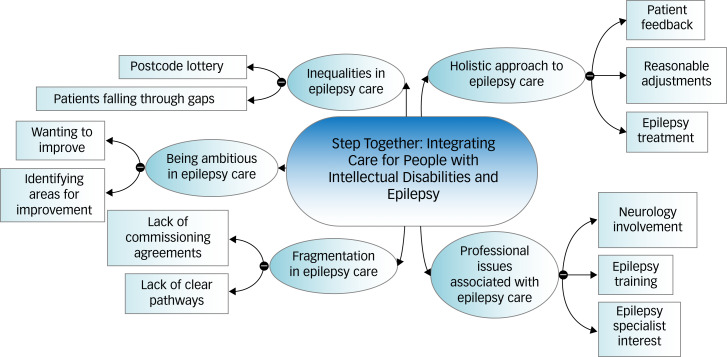
Thematic map of final themes generated by the reports.

## Data Availability

The data that support the findings of this study are available from the corresponding author, R.S., upon reasonable request.

## Appendix 1

### The Step Together Guidance

The Step Together guidance (https://www.bild.org.uk/wp-content/uploads/2020/11/Step-Together-17-November-2020-Download-Link-.pdf^23^) outlines the components of ideal care within a health and social care system. It allows comparison to identify gaps in care created by service non-alignment. It was developed from NHS England funding to address inequalities for PwID and epilepsy in England. Over 2 years (2018–2020), Step Together brought together PwID and epilepsy, their families, professional care providers, epilepsy and intellectual disability charities, expert clinicians in intellectual disabilities and epilepsy (neurologists, epilepsy nurses, neuropsychiatrists, psychiatrists working with PwID, school nurses, paediatric neurologists, paediatricians, etc.), NHS commissioners and social care professionals in a series of meetings, and used a modified Delphi process to summarise and present evidence. The final document has been supported and endorsed by the ILAE (British Chapter), the Royal College of Psychiatrists, epilepsy specialist nurses, the National Learning Disability Senate and various national charities working with people with epilepsy or intellectual disabilities. The ILAE has recognised Step Together as part of it meeting its curriculum requirement Learning Objective 6.1.

The purpose of the Step Together guidance was to facilitate the creation of capable communities in healthcare systems to support PwID with epilepsy. A ‘capable community’ consists of a broad spectrum of professionals from across the healthcare, social care and voluntary sectors, working toward the optimum safety, management and quality of life for each individual person. It ensures people receive the right input at the correct time, and that the person, carers and professionals receive the information that they need to maximise quality of care and life. Capable communities are based on the theory of communities of practice, and apply that theory to cross-team and cross-organisation collaboration to create learning and relationship building.

The Step Together toolkit, emerging as part of the Step Together guidance, is an evidence-based service self-assessment tool that allows healthcare providers to assess their service against the Step Together guidance. It is hosted by Epilepsy Action (https://www.epilepsy.org.uk/professional/step-together#row-fc-4). Both the Step Together guidance and toolkit were co-created with neurologists, psychiatrists, epilepsy nurses, primary care physicians, allied healthcare professionals, people with epilepsy and intellectual disability and their families and carers. A series of workshops were held across the UK where all the stakeholders came together to discuss what was necessary for quality holistic care for people with epilepsy and intellectual disability, and what an ideal service would look like. These discussions were analysed, themes identified and used to create the guidance, and subsequently the questions and domains included in the toolkit.

## Appendix 2

### Creation and validation of the Step Together toolkit

#### Identifying a need for a toolkit

The rationale and standards that the toolkit measures against can be found in the Step Together guidance. The concepts of the Step Together tool was laid out there and further highlighted in the seminar in epileptology addressing Learning Objective 6.1.4 of the ILAE Curriculum, ‘Demonstrate the ability to recognize and manage the special needs of persons with epilepsy’.^20^

Following its publication, the Step Together guidance document was promoted within healthcare systems through a series of online webinars with interested professionals and discussions with NHS leaders and commissioners. During this engagement, NHS regional leaders and commissioners of health and social care services were asked how the quality of what was commissioned and provided in their health economy could be systematically assessed against the guidance. As central planners, they wanted to be able to compare different services within the regions, so that efforts could be directed into improving services where the greatest inequities were apparent or could be evidenced. They suggested a survey or questionnaire could be used to identify and evidence areas in need of improvement before they would consider agreeing to invest time in workstreams. They reported that having the results of a survey or audit that could be presented in a workshop would focus minds on developing action plans to fully implement the guidance, and would justify the investment of time and resources into such plans.

Following this feedback, and similar feedback received during the webinars from healthcare professionals, it became clear that an integrated approach to quality improvement was needed. This would bring together system leads in local areas with clinicians who provide existing services to identify gaps in local provision. It was felt that a questionnaire-type toolkit that received input from the whole spectrum of health and social care professionals would best inform implementation. This would also improve uptake and adherence to the guidance, and assist commissioners in addressing gaps and inequalities in care by highlighting areas of need and giving recommendations on how to address them.

#### Key items for inclusion in the toolkit

The key elements that the guidance indicated should be part of safe care were identified and formulated into a set of questions. These questions were designed to be used by representatives from an ICS), health economy or key stakeholders in the provision of services to evaluate where local services met the guidelines and where they might collaborate to implement elements of the guidance not yet in place. These items were then tested and refined in a series of workshops with healthcare professionals.

##### Workshops

The online workshops brought together healthcare professionals involved in the care of people with intellectual disabilities and epilepsy across the UK. Four regions were involved in these workshops. These were:

- Cumbria, Tyne and Wear (with representation from Cumbria, Northumberland, Newcastle, North Tyneside, Gateshead, South Tyneside, Sunderland);
- Midlands (with representation from Birmingham, South Staffordshire, Shropshire, Coventry, Wolverhampton);
- Northern Ireland (with representation from the Belfast Trust);
- Oxfordshire (with representation from across the region).

The workshops had two purposes. The first was to discover what care looked like for each region, what they felt good care constituted and where they felt they had gaps in care or a need for improvement based on the Step Together guidance. The second purpose was to test and refine the questions identified from the Step Together guidance. The questions were tested to see whether they covered all aspects of care mentioned by participants, whether they were applicable and understandable to all regions, and whether they identified the gaps and areas in need of improvement that the attendees had identified themselves. Both of these purposes fed into the refinement of the toolkit questions by improving the questions already identified and adding additional questions to cover areas that had not been addressed. The guidance was broken down into sections to facilitate discussion by staff from different disciplines, to assess the extent to which current services met the guidelines.

General descriptions of the services provided in the area were given, and then the agreed questions were used to focus attendees on specific elements of the guidance. Attendees were asked to rate how well they felt their service was doing, using a simple rating scale of red, amber and green. When an element of care or management was substantially in place it was assessed as green, when there was a concern that there was a gap it was assessed as red and where plans were in place or implementation was patchy is was assessed as amber. These discussions were transcribed into four reports, one for each region. The reports identified the key themes from the discussions, which were used to inform the toolkit development.

##### Analysis

The analysis was a secondary thematic analysis of the four reports, using Braun and Clarke's^42^ reflexive thematic analysis six-step approach. An inductive approach was used.

There were six stages to the thematic analysis:

- Familiarisation with the data: the reports were read multiple times by the analyst, who was not present at the meetings and did not transcribe the reports, and therefore was not familiar with the data. This allowed for reduction of bias or suggestibility;
- Coding the data;
- Generating initial themes;
- Reviewing and developing themes;
- Refining, defining and naming themes;
- Producing the report.

Member checking, a full audit trail and triangulation were used to ensure the accuracy of the data analysis. Data analysis was checked by senior research authors.

#### Results

The analysis generated five main themes: holistic approach to epilepsy care, professional issues associated with epilepsy care, lack of clear service provision documents for planning care, inequalities in epilepsy care and being ambitious in epilepsy care (Fig. 2 and Table 8).

**Table 8:** Main themes, subthemes emerged from the supporting statements for the reports

Main theme	Subtheme
Holistic approach to epilepsy care Professional roles involved in epilepsy care Lack of clear service provision documents for planning care Inequalities in epilepsy care Being ambitious in epilepsy care	Patient feedback Reasonable adjustments Epilepsy treatment Neurology involvement Epilepsy training Epilepsy specialist interest Lack of commissioning agreements Lack of clear pathways Developing plans and policies Postcode lottery/different service set-ups across regions Patients falling through gaps Wanting to improve Identifying areas for improvement

##### Toolkit development and feasibility testing

Analysis of the themes emerging from the workshops illustrated that even in neighbouring places/teams activity, systems and the extent of implementation of the guidance and best practice varied. Tellingly, the experience for families could be very different if they moved a mile down the road.

The guidance and examples of good practice took time to work through, and in a 2 h workshop with people from different teams, typically 1 h was spent describing the different ways of working of each team. This suggested that the use of a tool would help to audit services and assess quality against the guidance in a structured way. These discussions also informed the wording of the toolkit questions to be as applicable as possible to all ways of working. It was agreed that where gaps were identified, a tool would make it clearer what type of new or enhanced services could be commissioned. It could also show if implementation of the guidance needed a quality improvement programme or a corporate work stream.

The questions that had been tested in the workshops were circulated to the attendees for comment. They were refined and expanded upon to become a benchmarking tool that would enable respondents’ assessments to be combined to give a picture of the quality of care across a local health and social care system. This would highlight areas for improvement and could inform action plans. Through evolving drafts, a digital tool was developed that collates and visualises responses to the questions, and this became the Step Together toolkit.

###### Testing the digital tool

The questions, now incorporated into a digital tool, were tested by a number of epilepsy and intellectual disability services within the UK. Fifteen services in total completed testing, comprising 13 services in England, one in Wales and one in Northern Ireland. A representative from each service, typically a specialist nurse, completed the toolkit on behalf of their service. They then gave feedback on how easy the toolkit was to use, any misunderstandings in question wording, how understandable and useable the results were, and what actions they would take based on their results. This feedback was used to finalise the wording of the questions and the presentation of the toolkit. It also informed training documents to support toolkit completion. The final version of the Step Together Toolkit has received input from a very wide range of healthcare professionals from across England, Wales and Northern Ireland, to ensure it reflects the needs and challenges faced by services across the UK.
