# Evidencing the challenges of care delivery for people with intellectual disability and epilepsy in England by using the Step Together toolkit – CORRIGENDUM

**DOI:** 10.1192/bjo.2024.828

**Published:** 2024-11-07

**Authors:** Tom Shillito, Lance Watkins, Hafsha Ali, Georgia Page, Angie Pullen, Sarah Mitchell, Ashok Roy, Arjune Sen, Michael Kinney, Rhys Thomas, Phil Tittensor, Manny Bagary, Arun Subramanium, Bridie Kent, Rohit Shankar

This article was originally published with co-author Georgia Page's name incorrectly given as ‘Georgina Page’.

This has now been updated and this corrigendum published.
