# Supplementary material for: Evidencing the challenges of care delivery for people with intellectual disability and epilepsy in England by using the Step Together toolkit
Source: BJPsych Open. 2024 Oct 28;10(6):e186. doi: 10.1192/bjo.2024.749 (PMC11698203; doi:10.1192/bjo.2024.749)
Supplement: Shillito et al. supplementary material 2 — Shillito et al. supplementary material [file S205647242400749Xsup002.pdf]

21 Prescott Street
London
E1 8BB

New Anstey House
Gate Way Drive
Leeds
LS19 7XY

30th May 2024

Dear British Journal of Psychiatry Open Review Panel,

RE: Evidencing the challenges of care delivery for people with intellectual disability and epilepsy in England using the Step Together toolkit

We are writing regarding the above manuscript, which was submitted to your journal recently. Epilepsy Action and Midlands and Lancashire Commissioning Support Unit were collaborators on this project, and both have staff who are authors on this paper. Following peer review, a concern regarding ethical approval has been raised with the authors, which we would like to address.

There was a concern that patients and carers had provided information which was used in this research and that ethical approval had not been sought. We would like to reassure the reviewers that no patient, carer or family data was provided to the authors or used directly in the research. The project did not recruit participants to take part or collect information from anyone based on:

a) their use of NHS or social care services
b) being a relative or carer of a service user

This research gathered data using a self-assessment benchmarking tool, called the 'Step Together toolkit'. This toolkit was completed by healthcare professionals, and asked only for the views of healthcare professionals regarding the services they provide.

Each individual Integrated Care System (ICS) taking part was advised to seek feedback from service users, families and carers when completing the Step Together toolkit, and to use that information to inform their responses. However, none of the questions in the toolkit are directed towards service users, carers or families. All questions asked were directed towards healthcare professionals working within the NHS. The input of service users, carers and families was suggested only to ensure the systems' responses would be fully informed by patient experience. The information they may have gained from those consultations was not directly inputted into the toolkit. The authors and their colleagues did not receive this information, or any information regarding consultations with service users, carers or families. There was not a requirement to know the detail from any consultations undertaken, discussion outputs or information given. Many of the ICS did not disclose whether they had undertaken such consultations, and those that did, did not disclose any of the contents of those discussions to the authors or their colleagues.

None of the authors or their colleagues received any information directly from service users. The only information received was aggregate information in the form of the toolkit responses. This data is quantitative and appears as answers to multiple choice questions, mostly yes/no or agree/disagree style questions. None of the questions ask directly about patient experience. There is one section on service user engagement, and the questions in this section ask about how often patients, carers and families are involved in decision making, and whether they could give feedback or receive a response. This section does not ask questions of service users directly.

We would like to clarify that the authors and their colleagues did not receive any confidential or identifying information about patients, service users, carers or families. All information from non-healthcare professionals was gathered and used by staff within each ICS which sits outside the ethical requirements of the project.

We hope this has reassured you that ethical approval was not required for this research. If you have any further questions, please do not hesitate to contact us.

Yours sincerely,

Alison Fuller
Director of Health Improvement and Influencing
Epilepsy Action
afuller@epilpesy.org.uk

PKHundal

Pardip Hundal
Assistant Director for Quality Improvement and Health Inequalities and Deputy Programme Director
Learning Disability, Autism/SEND Programme
Midlands – NHS England
Pardip.hundal@nhs.net
