# Supplementary material for: Evidencing the challenges of care delivery for people with intellectual disability and epilepsy in England by using the Step Together toolkit
Source: BJPsych Open. 2024 Oct 28;10(6):e186. doi: 10.1192/bjo.2024.749 (PMC11698203; doi:10.1192/bjo.2024.749)
Supplement: Shillito et al. supplementary material 4 — Shillito et al. supplementary material [file S205647242400749Xsup004.docx]

Supplementary information 2

Feedback from four of the Step Together Chapions in Midlands Region on use of *Step Together Toolkit*

1. “*To continue to improve capability and capacity for commissioners, epilepsy is now a distinct workstream in our LD/A planning. [Our] LeDeR Governance Panel approved “Epilepsy” as a local variation for focussed LeDeR Reviews. The Task and finish Group [established to complete the toolkit] will become a forum with strategic oversight of pathway scoping and commissioning intentions for our citizens with intellectual disability and epilepsy. The initial webinar provided a platform to build on for the future, with [our] ICB now hosting quarterly webinars, with a shared focus on ‘hot topics’ from our LeDeR programme, commencing with SUDEP in Q1 2023/2024. These system commitments will help reduce the risk of health inequalities and premature mortality for our people with ID and epilepsy.*”
2. “*The toolkit cannot be completed without input from a very wide range of stakeholders, and even before the toolkits had been returned we could see the benefits those conversations were having. This approach required providers to be aware of and evaluate against the patient journey, the range of services this population need to support them from both a health and social care perspective, the number of people involved in their care, and the conflicting needs of each service. It also highlighted the needs this population require outside of each individual professional’s speciality.*”
3. “*The process to undertake this self-assessment has created not only ownership of this issue but helped to create advocates for change. The product of all stakeholders being systematically involved in this work has enabled systems to collectively review and benchmark how all services can best meet the needs of this population; to identify gaps, variation, challenges and to focus their service improvement plans on agreed areas of impact for service users.*
4. *“Client and stakeholder expectations have been exceeded. Recommendations to partners were specific and gave a clear steer where differences could be made for patients’ safety in a neutral non-judgemental fashion, providing a strong platform on which to go forward with a “can do” purpose. This work has left us not only with quantifiable actions, made populations visible and given system ownership of the service issues faced in this critical area, but has created an advocacy for change and a social movement to improve the lives for whom we absolutely need to, and can, make a difference.”*
